# Tobacco-Driven Oral Cancer in India: Clinicopathological Correlates and Prevention Priorities

**DOI:** 10.7759/cureus.89780

**Published:** 2025-08-11

**Authors:** Mohammad Saquib ali, Ashish K Chaudhary, Priyesh Shukla, Neetu Purwar, Pramod K Singh

**Affiliations:** 1 General Surgery, Ganesh Shankar Vidyarthi Memorial Medical College, Kanpur, IND; 2 General Surgery, Government Medical College, Azamgarh, IND; 3 Pathology, Ganesh Shankar Vidyarthi Memorial Medical College, Kanpur, IND; 4 Radiation Oncology, Ganesh Shankar Vidyarthi Memorial Medical College, Kanpur, IND

**Keywords:** bidi, gutka, oral cavity cancer, south asia, squamous cell carcinoma, tnm staging

## Abstract

Background: Oral cavity cancer (OCC) is a significant public health concern in India, where tobacco use contributes to aggressive disease patterns, mainly smokeless tobacco. This study evaluated the clinicopathological profile of OCC patients, focusing on the influence of gutka, bidi, and cigarette use, as well as age distribution and TNM staging.

Methods: A retrospective observational study was conducted on 200 biopsy-confirmed OCC patients at a tertiary care centre. Data included demographics, tobacco habits (gutka, bidi, cigarettes), and tumor staging (TNM classification). Statistical analyses were performed using SPSS software, version 29 (IBM Corp., Armonk, NY), with significance set at p < 0.05.

Results: The mean age of patients was 47.1 years (SD = 12.0), with 78.3% (n = 65) being male. Gutka use was the most prevalent (54.2%, n = 45), followed by bidi (25.3%, n = 21) and cigarette smoking (16.9%, n = 14). Stage IV disease was observed in 60.2% (n = 50) of patients. T4 tumors were present in 43.4% (n = 36), and N2 nodal involvement in 31.3% (n = 26). Gutka use was associated with advanced T4 tumors (adjusted OR = 2.8; 95% CI: 1.6-4.9; p = 0.002), while bidi smoking predicted nodal metastasis (N2 stage: OR = 1.9; 95% CI: 1.1-3.4; p = 0.03).

Conclusion: OCC in India predominantly affects middle-aged males with a history of gutka or bidi use. Our findings mandate screening for 'gutka', a smokeless tobacco preparation, and 'bidi' (local cigarette) users, and strict enforcement of tobacco bans to curb advanced-stage diseases, especially focusing on regions prone to tobacco exposure in different forms, especially smokeless tobacco called 'gutka', widely used in Kanpur, India.

## Introduction

While there are declining global oral cancer rates, India faces a worsening epidemic fueled by smokeless tobacco. This preventable crisis demands attention [1]. It ranks among the top 10 most common cancers in South-Central Asia, with India, a developing nation, contributing nearly 20% of the global Oral Cavity Cancer (OCC) burden [2]. The high incidence seen in this region of the world is strongly associated with the widespread consumption of tobacco in various forms, including smoked ('bidi', cigarettes) and smokeless ('gutka', 'kheni', betel quid) products [3]. Despite recent advances in diagnostic and therapeutic modalities, the five-year survival rate for OCC remains dismal at approximately 50-60%, primarily because of late-stage diagnosis and high rates of recurrence among the population [4].

Primary risk factors for OCC include tobacco use, alcohol consumption, betel quid chewing, poor oral hygiene, and human papillomavirus (HPV) infection [5]. In India, smokeless tobacco products such as 'gutka' (a mixture of areca nut, slaked lime, and tobacco) and 'khaini' (a powdered tobacco product) are widely consumed, particularly among men who belong to low socioeconomic groups [6]. Studies indicate that 'gutka' chewers have a 20-fold increased risk of developing OCC as compared to non-users [7]. Similarly, 'bidi' smoking, a form of tobacco consumption in India for ages, has been linked to a higher incidence of oral squamous cell carcinoma (OSCC) due to its high tar and carcinogen content [8]. 

OCC predominantly presents as squamous cell carcinoma (90% of cases), with the buccal mucosa and tongue being the most involved sites [9]. Early-stage OCC (Stage I-II) often presents as non-healing ulcers or leukoplakia, while advanced stages (III-IV) present with pain, trismus, dysphagia, and cervical lymphadenopathy [10]. Tumor staging, which is based on the TNM classification system, is one of the critical prognostic factors, with T4 tumors and nodal metastases (N+) indicating poorer outcomes [11]. 

This study aims (a) to examine the association between tobacco habits ('gutka', 'bidi', 'khaini', and cigarettes) and OCC, (b) analyse the clinicopathological characteristics and TNM staging of OCC in a tertiary care setting, and (c) identify high-risk populations for targeted screening and intervention.

## Materials and methods

This study employed a retrospective observational design to analyze 200 consecutive cases of biopsy-proven oral cavity cancer (OCC) treated at the Department of Surgery and Department of Oncology (JKCI), Ganesh Shankar Vidyarthi Memorial (GSVM) Medical College, Kanpur, between June 2023 and December 2024. The study protocol adhered to STROBE guidelines for observational studies and received ethical approval from the Institutional Ethics Committee of GSVM Medical College (EC/BMHR/2024/41, dated 16-01-2024) in compliance with the Declaration of Helsinki. Standardized proformas were used to record demographics, tobacco/alcohol habits (quantified as pack-years for 'gutka'/'bidis' and grams/day for alcohol), and clinicopathological features. Data sources included electronic medical records (EMRs) and structured interviews.

The study population included adult patients (age ≥18 years) with histologically confirmed OCC who provided written informed consent. Patients unwilling to participate or not interested in replying to the questionnaire were excluded. Data collection encompassed three domains: demographic characteristics, risk factor assessment, and clinicopathological evaluation. Demographic variables included age, gender, and occupation, while risk factor analysis focused on tobacco habits ('gutka', 'bidi', 'khaini', cigarettes), alcohol consumption quantified in grams per day, and betel quid chewing history. Clinical examination documented tumor site (buccal mucosa, tongue, floor of mouth, lips, hard palate), size, morphology, and symptoms such as trismus, dysphagia, and bleeding.

Pathological staging followed the American Joint Committee on Cancer (AJCC) 8th edition TNM classification system, with tumors categorized by depth of invasion (T-stage), nodal involvement (N-stage), and distant metastasis (M-stage). Histopathological grading classified tumors as well, moderately, or poorly differentiated squamous cell carcinomas. Statistical analysis was performed using SPSS software, version 29 (IBM Corp., Armonk, NY). Descriptive statistics summarized continuous variables as mean ± standard deviation and categorical variables as frequencies with percentages. The Chi-square test (with degrees of freedom and effect size reported) assessed associations between categorical variables, while logistic regression modeled risk factors for advanced-stage disease. A two-tailed p-value <0.05 was considered statistically significant.

## Results

Demographics and risk factors

The study included 200 patients with biopsy-proven oral cavity cancer. The mean age at presentation was 47.0 years (SD = 12.0 years), with a peak incidence observed in the 31-50-year age group (56.5%, n=113). A significant proportion of patients (18.0%, n=36) were younger than 40 years, indicating a trend toward earlier onset of disease. The cohort showed marked male predominance (86.5%, n=173 male vs 13.5%, n=27 female), reflecting the higher prevalence of tobacco use among men in this region. Socioeconomic analysis using the modified Kuppuswamy scale revealed that most patients belonged to lower-middle (45.0%, n=90) and lower (38.0%, n=76) socioeconomic classes. Table 1 describes the number of subjects in different age groups.

**Table 1:** Demographics

AGE	No. Of Cases
21-30	12
31-40	60
41-50	53
51-60	46
61-70	21
71-80	8
Mean	33.3

Tobacco and alcohol use 

'Gutka' chewing was the most prevalent tobacco habit (80.0%, n=160), followed by 'bidi' smoking (50.5%, n=101) and cigarette use (47.5%, n=95). 'Khaini' use was reported in 22.5% (n=45) of cases. 'Gutka' chewers demonstrated significantly higher odds of developing T4 tumors (OR = 2.8; 95% CI: 1.6-4.9; p=0.002; χ² = 12.4, df=1, φ=0.25). 'Bidi' smokers showed increased risk of nodal metastasis (N2 stage: OR = 1.9; 95% CI: 1.1-3.4; p=0.03; χ² = 6.2, df=1, φ=0.18). Alcohol consumption was reported by 62.0% (n=124) of participants, with combined tobacco-alcohol use significantly associated with advanced-stage disease (OR = 3.2; 95% CI: 1.9-5.5; p<0.001). Table 2 describes the tobacco chewing ('gutka') pattern among males and females.

**Table 2:** Tobacco ('gutka') chewing

Gender	Tobacco ('Gutka') Chewing	N	Mean	SD	p-value
Male	Yes	138	46.6	11.8	0.005
	No	35	48.4	12.8	0.337
Female	Yes	22	51.0	13.9	0094
	No	5	48.8	13.1	0.862

Tumor characteristics

The buccal mucosa was the most frequent tumor site (67.5%, n=135), followed by the lateral tongue (21.5%, n=43), floor of mouth (6.5%, n=13), hard palate (2.5%, n=5), and lips (2%, n=4). Advanced disease at presentation was common, with 53.0% (n=106) of cases classified as Stage IV. T4 tumors accounted for 45.3% (n=91) of cases, while N2 nodal involvement was present in 48.2% (n=84). Poorly differentiated tumors (11.5%, n=23) were more prevalent in younger patients (<40 years) and showed a stronger association with nodal spread (p=0.041).

Clinical presentation

The majority of patients presented with pain (74.0%, n=148) and trismus (55.0%, n=110), particularly those with posterior buccal mucosa involvement. Other symptoms included bleeding (20.0%, n=40, p=0.023), halitosis (14.5%, n=29), and dysphagia (11.5%, n=23). Trismus and halitosis were significantly associated with Stage IV disease (p<0.001), suggesting their utility as clinical markers of advanced local invasion.

Histopathological findings

Well-differentiated squamous cell carcinoma (52.0%, n=104) was the most common histological subtype, followed by moderately differentiated (36.5%, n=73) and poorly differentiated tumors (11.5%, n=23). Poor differentiation correlated with younger age (p=0.038) and nodal metastasis (p=0.041).

Multivariate logistic regression demonstrated that gutka use remained an independent predictor for T4 tumors (OR = 2.8; 95% Cl: 1.6-4.9, p=0.002), while bidi smoking was independently associated with N2 nodal metastases (OR = 1.9; 95% Cl: 1.1- 3.4, p=0.03). Combined tobacco and alcohol use significantly increased the odds of advanced-stage disease (OR = 3.2; 95% Cl: 1.9-5.5, p<0.001).

## Discussion

Tobacco as the dominant risk factor

Our data reveal 'gutka' and 'bidi' as independent drivers of advanced OCC, creating a window for early intervention in South Asia's youth-dominated epidemic. The finding that 'gutka' chewers (80.0%, n=160) had 2.8X higher odds of T4 tumors (95% CI: 1.6-4.9; χ²=12.4, df=1, p=0.005) confirms the aggressive local invasion pattern associated with smokeless tobacco. Similarly, 'bidi' smoking, prevalent among low-income populations owing to its affordability, was associated with a higher rate of nodal metastasis (N2 stage). Unlike cigarettes, 'bidis' are hand-rolled with coarse tobacco and deliver more tar, carbon monoxide, and nicotine per unit, making them even more hazardous. Our data align with a recent study [8], which reported increased lymph vascular invasion and perineural spread in those patients who were chronic 'bidi' users. 

The synergistic effect of alcohol and tobacco further exacerbates the risk of OCC. Alcohol acts as a solvent to enhance mucosal permeability and facilitates the absorption of carcinogens. In our study, the combined use of alcohol and tobacco significantly increased the likelihood of advanced-stage presentation (odds ratio (OR)=3.2; p<0.001), echoing the similar findings of a study [12] that reported multiplicative interactions between these substances in head and neck cancers. 

Age and gender disparities

The mean age of patients was 47 years, with more than half being between 31 and 50 years, suggesting a notable trend of OCC affecting individuals in their prime working years. This trend reflects early exposure to carcinogenic habits, particularly gutka, which are often initiated in adolescence owing to peer influence, lack of awareness, and aggressive marketing. Unlike Western populations, where OCC typically peaks in the sixth or seventh decade of life, Indian patients are presenting a decade earlier, as observed in several studies [6,13]. A stark male predominance (86.5%) was evident in our cohort, which mirrors national statistics and underscores the higher prevalence of tobacco use among Indian men. Cultural and societal norms often discourage women from smoking or chewing tobacco publicly, which may contribute to underreporting in the female population. Nonetheless, the increasing use of smokeless tobacco among rural and tribal women warrants closer epidemiological scrutiny. 

Clinicopathological correlations

The buccal mucosa was the most commonly affected site (67.5%), consistent with regional chewing practices in which the quid is habitually retained in the buccal sulcus. This contrasts with data from Western countries, where the tongue and floor of the mouth are more frequently involved, and are often linked to alcohol consumption, cigarette smoking, and HPV infection [4]. The high rate of T4 tumors (45.3%) and Stage IV presentation (53%) at diagnosis reflects delayed healthcare-seeking behavior, lack of routine oral screening, and limited access to specialized care. Trismus, found in 55% of patients, was significantly associated with posterior buccal mucosa involvement and muscle infiltration, serving as a clinical marker for advanced local disease. Although less frequent, halitosis and bleeding have been linked to tumor ulceration and necrosis. These symptoms, when recognized early, could prompt early clinical evaluation and biopsy, potentially improving prognosis. 

Histologically, most cases were well to moderately differentiated squamous cell carcinomas, aligned with patterns observed across Indian cohorts. However, poorly differentiated tumors, although less common (11.5%), were more likely to occur in younger patients and had a higher likelihood of nodal metastasis. This may indicate a more aggressive tumor biology when we talk about younger populations, as previously reported in another study [13]. 

Public health implications 

We propose a three-tier clinical strategy: (1) community screening for 'gutka' users with oral examinations, (2) nodal ultrasounds for 'bidi' smokers, and (3) school-based prevention targeting adolescents. Although 'gutka' has been banned in several Indian states, its enforcement remains inconsistent, and black-market sales continue to be unabated. Targeted legislative efforts, including plain packaging, warning labels, taxation, and crackdowns on illicit sales, are essential to reduce accessibility and consumption. 

Screening programs should prioritize high-risk populations, especially men aged 30-50 years with a history of smokeless tobacco use. Community healthcare workers play a pivotal role in oral screening, particularly in rural and underserved areas. The inclusion of oral examinations in routine health check-ups at primary care levels could facilitate early detection of leukoplakia, erythroplakia, and early-stage malignancies. Cessation support must also be integrated into primary healthcare by combining pharmacological interventions (e.g., nicotine replacement therapy) with behavioral counselling. School-based education programs focusing on the long-term consequences of tobacco use could help reduce initiation rates among the youth. 

Comparison with global literature

The finding of this study shows similar results to regional data from Pakistan [14], Bangladesh [15], and Sri Lanka [16], where smokeless tobacco use is endemic. However, they diverge from trends in Western nations, where HPV-positive oropharyngeal cancers are increasing owing to changing sexual behaviors and HPV type 16 prevalence. The absence of HPV screening in our cohort is a limitation, although HPV is less commonly associated with anterior oral cavity cancer. Mechanistic studies show gutka's arecoline and tobacco-specific nitrosamines induce epithelial-mesenchymal transition through chronic inflammation [17]. The buccal mucosa predominance (67.5%, n=135) directly reflects the quid-retention habit unique to South Asian chewers [18].

Study limitations

Although these findings provide valuable insights, this study has certain limitations. As this was a single-center study, the results may not be generalizable across other regions of India with different cultural and consumption patterns. Second, self-reported data on tobacco and alcohol use may be prone to recall and social desirability biases, especially among female participants. Third, the absence of longitudinal follow-up data limited our ability to correlate clinical staging with survival outcomes.

## Conclusions

Oral cavity cancer (OCC) remains a formidable public health challenge in India, where the burden is disproportionately high owing to the pervasive use of tobacco in both smoked and smokeless forms. This study highlights a significant association between gutka and bidi consumption and the development of advanced-stage OCC, with over half of the patients presenting with Stage IV disease and a substantial proportion showing nodal metastasis at diagnosis. The predominance of buccal mucosa involvement further underscores the localized effect of carcinogens from smokeless tobacco retained in the oral vestibule for extended durations. 

Public health strategies must prioritize prevention and early detection, which includes enforcing bans and strict regulations on the production and sale of 'gutka' and similar carcinogenic products. Educational campaigns to raise awareness of the early signs of OCC and the dangers of tobacco use target both urban and rural populations. School-based programs to prevent initiation of tobacco use among adolescents. Cessation support services, including behavioral counselling and pharmacotherapy, should be widely available and integrated into primary care settings. Future research should also explore molecular and genetic biomarkers that can predict disease progression or therapeutic responses, offering a path toward personalized treatment strategies. This study adds to the growing body of evidence that tobacco, especially smokeless tobacco, particularly 'gutka', plays a central role in the etiology and progression of OCC in India. A comprehensive, multidisciplinary approach encompassing policy, prevention, screening, and treatment is essential to curb this preventable cancer epidemic and improve patient outcomes.
